# The constraints and driving forces of oasis development in arid region: a case study of the Hexi Corridor in northwest China

**DOI:** 10.1038/s41598-020-74930-z

**Published:** 2020-10-19

**Authors:** Qiang Bie, Yaowen Xie

**Affiliations:** 1 School of Earth and Environment Science, Lanzhou University, Lanzhou, 730000 PR China; 2 Faculty of Geomatics, Lanzhou Jiaotong University, Lanzhou, 730070 PR China; 3 The Key Laboratory of Western China’s Environmental Systems, Ministry of Education, Lanzhou, 730000 PR China; 4 State Key Laboratory of Resources and Environmental Information System, Beijing, 100039 PR China

**Keywords:** Ecology, Ecosystem ecology, Climate and Earth system modelling

## Abstract

The oasis, a special landscape with the integration of nature and humanity in the arid region, has undergone an enormous transformation during the past decades. To gain a better understanding of the tradeoff between economic growth and oases stability in the arid land, we took the oases in the Hexi Corridor as a case to explore the constraints of oases development and the driving factors of oases expansion. The dynamic changes and spatial distribution patterns underwent by the oases were examined using multispectral remote sensing imagery. The constraints of oasis development in arid land were investigated by the grid-transformed model, as well as the index system of driving forces was analyzed using the grey incidence model based on the data from statistics yearbooks. The oasis area in the Hexi Corridor had tremendous changes expanded 40% from 1986 to 2015, the stable oasis area was 9062 km^2^, while the maximum area reached 16,374 km^2^. The constraints for oases of topography, hydrology and heat condition are as follow: The elevation of oasis ranged from 1000 to 1800 m, peaked in 1500 m; the slope of oasis distribution was flatter than 3 degrees; the aspect of oases on slope land concentrated in northeast and north, accounting for more than 60%. The main driving forces of oasis spatial expansion in the arid region were population, water resource, economy, policies, and other factors. These results are expected to (1) improve the rationality of oasis development, and (2) promote the sustainable planning and management of oases in the arid land.

## Introduction

Land use and land cover (LULC) change and its ecological effects have attracted worldwide attention since the 1990s^1–4^. Increased efforts have been devoted to investigating the pattern, process, trend, and driving forces of LULC change and its ecological responses. Arid and semi-arid land take up more than 30% of the land surface in global^5^ and 22% of the land surface in China^6^. Due to climate change, the unreasonable artificial activities, and the character of its own vulnerability, these regions suffered a large impact, thus becoming more sensitive to changes in environmental conditions^7–9^. Although the oases area in China takes up around 5% of the total arid and semi-arid area, the most population (90%) and social wealth (95%) in this area are concentrated in oases^10,11^. Therefore, Oasis, which is the special landscape of integration of nature and humanity in the arid region and is also the quintessence of the arid region, has become the focus of study. Besides, knowledge of the oasis changes and its constraints and driving forces is critical to promoting the sustainable management of oases.

Hexi Corridor is located in the northwest of China, composed of three inner river basins named Shiyang River Basin (SYRB), Heihe River Basin (HHRB), and Shule River Basin (SLRB). However, due to the disorganized, ineffective exploitations in the past decades, the ecological degradation including but not limited to the land desertification, soil salinization, declining in the groundwater table, and vegetation coverage decreases, became the most severe environmental issues, which refers to a deterioration in the functioning and productivity of an oasis ecosystem^12–15^. Zhang and Xie^15^ reported that from 1986 to 2015, the positive changes of oases in Dunhuang in SLRB dominated the overall change trend, where the observed changes were mainly distributed at the periphery of artificial oases located outside the reserve. Xie, et al.^16^ reported that the total area of oases in HHRB dramatically expanded by 60% from 1963 to 2013. Several attempts have been conducted to analyze the distribution and the changes of oases in the individual basin, e.g., SLRB^15^, the HHRB^16^, the SYRB^17,18^. The previous investigations of oasis changes mainly focused on a single watershed or even a smaller area, such as part of a watershed^18^ in these arid regions.

Recent studies on the mechanism of oasis changes in arid land mainly focused on influence of water scarcity on oasis development in HHRB^19,20^, dynamic mechanism of urban expansion in Hexi Corridor^19^, natural restrictions of oasis existence, and driving forces of land use/land cover change in the northwest of China^21^. Zhou et al.^19^ took the HHRB in Hexi Corridor to analyze the changes in oasis dynamics and their driving forces and concluded that the key driving force of the oasis expansion was sufficient irrigation water guaranteed by excessive consumption of groundwater and surface water and water conservation efforts. Besides that, the human driving factors such as population and economy increasing also contributed to oasis expansion, and soil fertility and the groundwater depth are the main natural restrictions of oasis expansion^21,22^. There are still, however, information gaps. First, Recent studies on the mechanism of oasis change driving force in arid land mainly focused on the qualitative analysis rather than quantitative analysis and analysis of factors independently, not comprehensively. Second, the constraints are the basis of oasis distribution and the driving forces are the causes the oases to expand or shrink. Available analyses are not sufficient to quantificationally depict the natural restrictions of oasis development in arid regions in the long past. In other words, the issue of assessing the potential distribution of oasis, the area where oasis could develop, has not received recognition from many researchers.

The oasis change was caused not only by the changes in natural conditions including climate, water resources but also by interference with human activities. Consequently, it is urgent to provide detailed and quantitative information regarding the driving forces of the oasis change in past thirty years, to deal with the more frequent and intensive interventions between climate and humans in future^23,24^.In the light of climate and human interventions and the accelerating pace of rural revival^25^, it is critical to examine the changes of oases in past thirty years within which the oases changed greatly and to depict the restrictive conditions and driving forces of oasis existence and development. In this work, we have three specific research objectives to: (1) analyze how have the oases changed in past thirty years in the Hexi Corridor and; (2) quantitively analyze the constraints of oases distribution in Hexi Corridor; and (3) evaluate the driving forces of oasis change in an arid and semi-arid land.

## Results

### Characteristics of oasis change in the Hexi Corridor

#### Oasis area variation at the river basin and county scales

**Figure 1:**
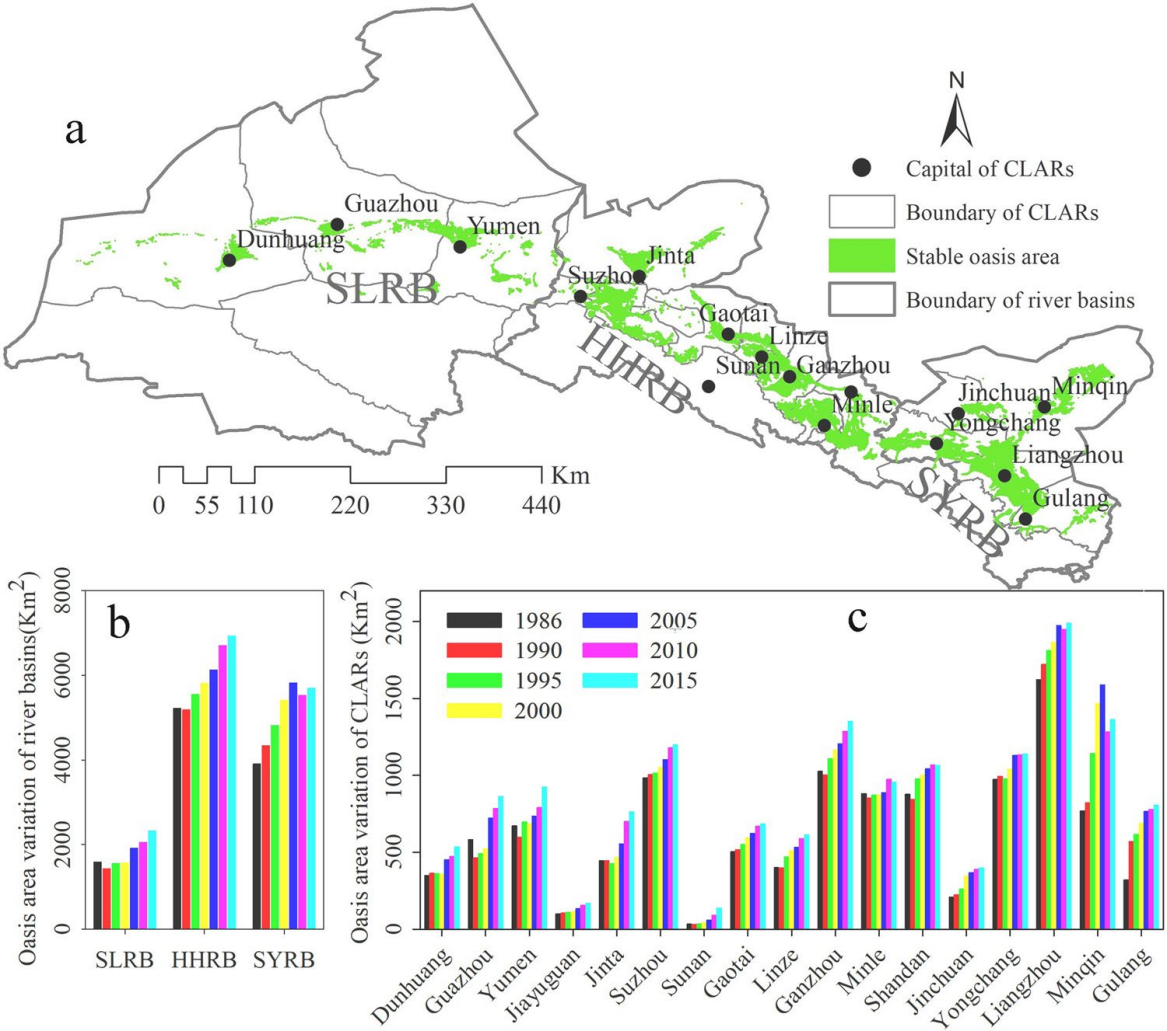
Variation of oases area in three river basins of the Hexi Corridor during the past thirty years. (**a**) was generated using ArcGIS 10.3, www.esri.com.

The distribution of stable oasis in three river basins and seventeen administration regions is shown in Fig. 1a. The total oasis area in the Hexi Corridor has increased from 10,707.7 km^2^ in 1986 to 14,950.1 km^2^ in 2015 (Fig. 1b), with an increase factor of 1.4 from the start to the end years and an average annual increase of 140 km^2^. At the river basin scale, the HHRB has the largest oasis area with 47% of the total oasis area, followed by SYRB with 40%. The SLRB charactered by drier environments has the least oasis area of 13%. The oasis change types in the Hexi Corridor over the last 30 years are mainly “expansion”, which is supplemented by “retreating” (Fig. 1b). The oasis area variation of administration regions during the past thirty years is shown in Fig. 1c. It is observed that the variation tendency of the oasis area at administration regions scale was the same as that on the river basin scale. The oasis areas in Liangzhou District, Ganzhou District, Minqin County, Yongchang County, Suzhou District, and Shandan Country were more than 1000 km^2^ in most time. Conversely, the oasis area in Jiayuguan District and Sunan County was less than 200 km^2^.

#### The stable oasis and maximum oasis distribution

The stable oasis was extracted from the area where the oasis exists in all seven periods, and the maximum oasis area was depicted from the area where the oasis existed once in the past thirty years. It can be seen that the stable oasis area is 9062 km^2^, while the maximum oasis area reaches 16,374 km^2^, which is almost two times larger than that of the stable oasis.

**Figure 2:**
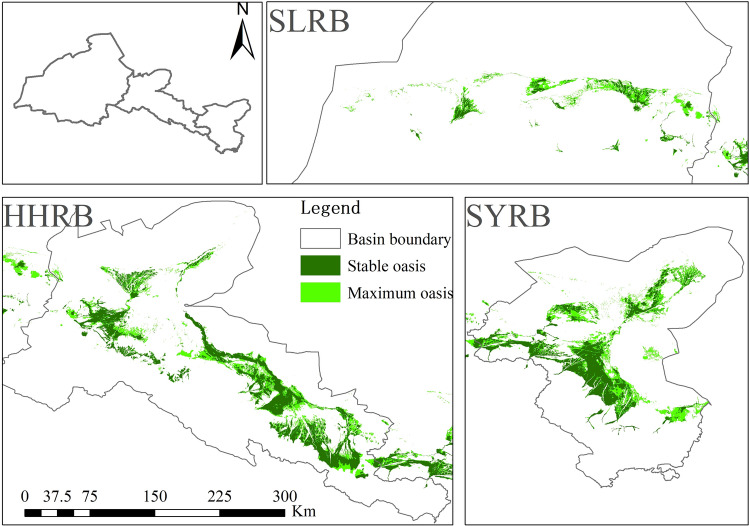
The distribution of stable oases and maximum oases in the Hexi Corridor. The map was generated using ArcGIS 10.3, www.esri.com.

The stable oases distribute in alluvial and pluvial fans, the river plains in middle reaches, and the catchment area in the lower reaches (Fig. 2). The maximum oases extended from the stable oases, which mainly located at the edges of the alluvial–proluvial fans, low-lying areas next to rivers and ditches, and the oases-deserts ecotone.

### The constraints of oasis development

#### Geomorphological characteristics of oasis distribution

**Figure 3:**
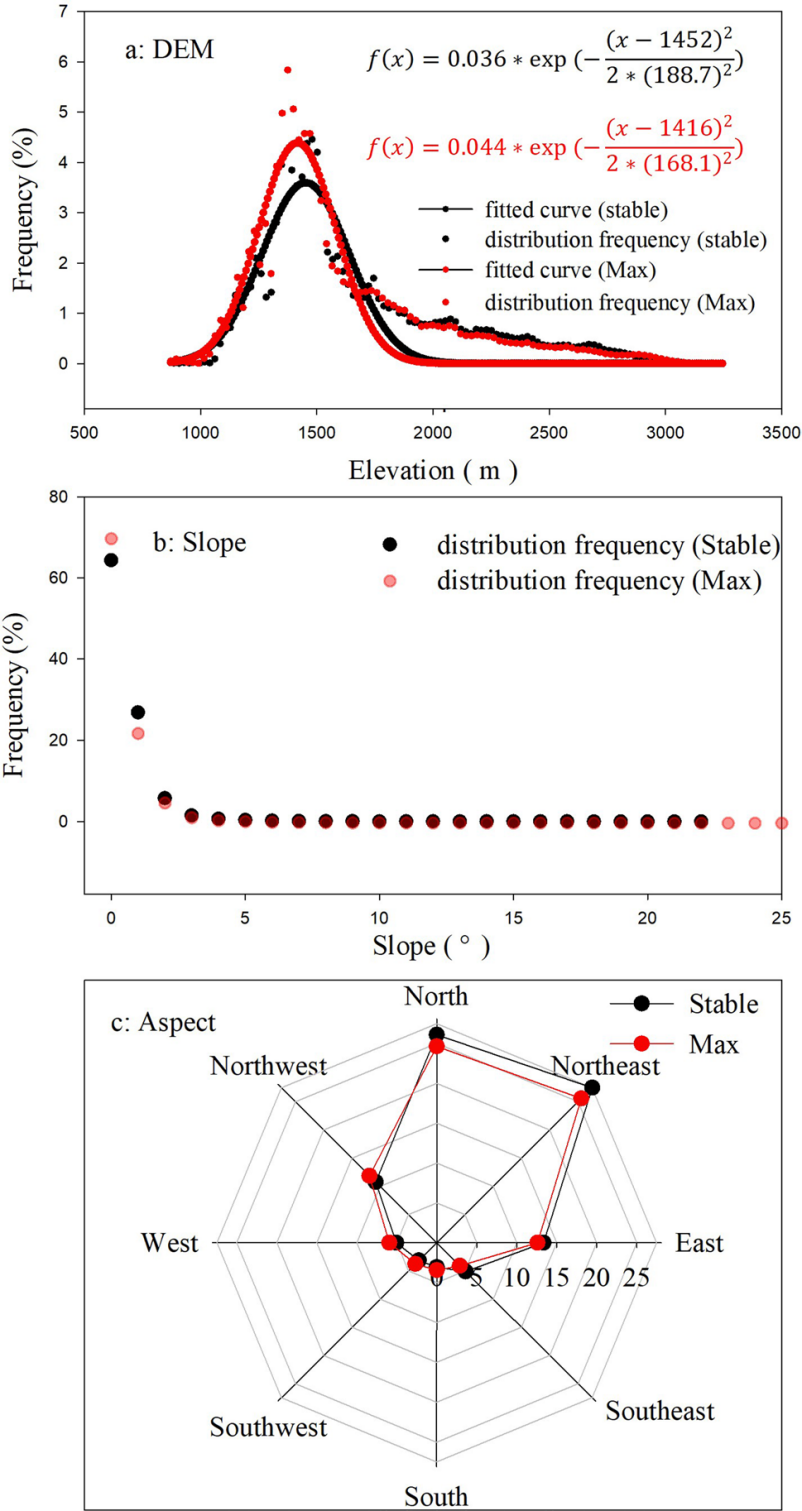
Topographic characteristics of oasis distribution: (**a**) the elevation, (**b**) the slope, and (**c**) the aspect distribution frequency of stable and maximum oases. (the black dot represents the distribution frequency of stable oasis, the red dot represents the distribution frequency of maximum oasis, the black dotted line is the fitted curve of the distribution frequency of stable oasis, and its formula is the black one, the red dotted line is the fitted curve of the distribution frequency of maximum oasis, its formula is the red one).

The geomorphological conditions, formed in the geological history period, is critical for the process of oases development. To investigate the possible relationship between limiting factors and oasis distribution, the distribution frequency, which is the ratio of number in specific condition among all oasis raster number, was introduced and the scatter plots and normal distribution fitting curves were plotted. Figure 3a shows the altitude of the oasis is mainly between 1000 m that is near the lowest value in the study area to 2500 m. The elevation of stable (maximum) oasis peaks in 1500 (1450) m, and accounted for 3.5% (4.5%), which suggests that when oases expand, they tend to occupy the lower elevation. The oases are mainly located in the plains along rivers or irrigation canal systems where slopes flatter than 5° (Fig. 3b), most of them are located in the level ground with a slope flatter than 3°. The area of the stable and the maximum oases located in flat place (slope = 0) account 64% and 76%, respectively, which indicated that the oasis expansion mainly occurs on flat ground. The analysis of the oasis on eight slopes shows that the majority of the slope oasis is concentrated in the north slope and the northeast slope, accounting for about 60%, while the east slope and the northwest slope also have a part, accounting for 30% (Fig. 3c). The aspect of slope oasis expansion mainly takes place in sunny slope (Northwest, West, Southwest, south, southeast), which due to that almost all of the shady slope has been covered by oasis. On the contrary, there are many deserts in sunny slope, as long as the necessary moisture conditions will be occupied by the oasis. The different aspects result in varying amounts of solar radiation, which affects evapotranspiration and consequently water balance in the soil. More specifically shady aspects have more moisture for vegetation growth due to less evapotranspiration, on the other hand, sunny aspects experience potentially higher rates of evapotranspiration, supporting less moisture for vegetation growth^26^. The fitted normal distribution formula of DEM frequency for stable and maximum oases are given in Fig. 3a, the fits reached a significant level (*p* < 0.001).

#### Hydrology restriction of oasis distribution

**Figure 4:**
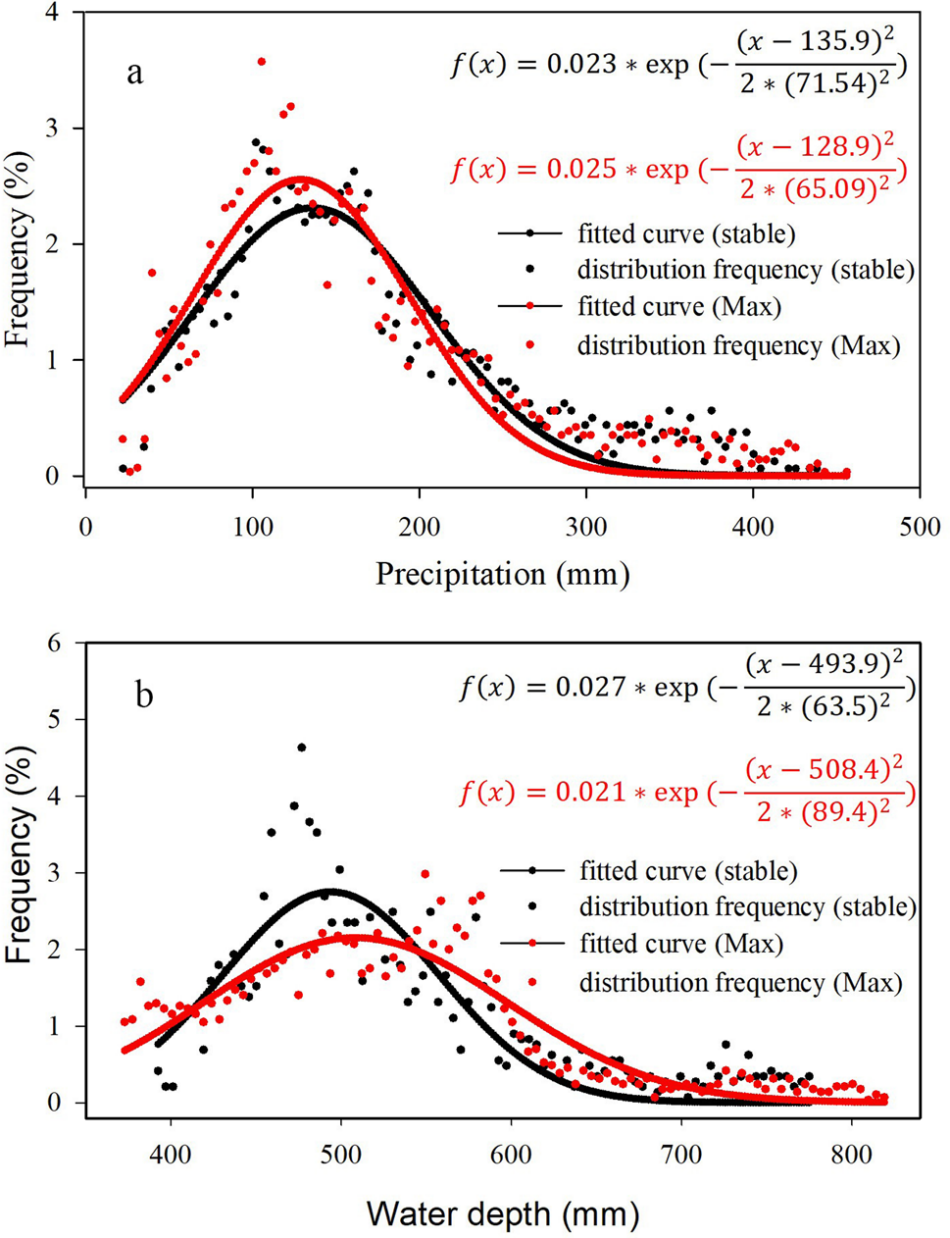
The distribution frequency of (**a**) precipitation and (**b**) water depth in the stale and maximum oasis (the legend is the same as Fig. 3).

The distribution frequency of precipitation in stable oasis and the maximum oasis was obtained by superposition analysis of gridded precipitation and oasis distribution. The most stable oasis distributes in the area where the precipitation is between 50 and 300 mm (Fig. 4a), which is limited by the lack of precipitation in the arid region. In fact, such low rainfall is not enough to sustain vegetation^27^. Plant growth and production are closely tied to shallow hydrologic systems, which is almost all of this from upstream runoff in an arid land. Therefore, the water depth, the sum of precipitation and the available runoff on grid-scale, was induced to examine the impact of hydrology on oasis development. Both the stable and maximum oasis located in the area of the water depth is larger than 400 mm (Fig. 4b). Moreover, the fit curve of maximum oasis is flatter than that of the stable oasis, which suggests that when oases expand, they tend to occupy lower water depth where the land cover was desert due to water shortage. The fitted normal distribution formulas of precipitation and available water depth for stable and maximum oases were shown in Fig. 4, all the fits passed the test (*p* = 0.001). We suggest that the oasis developed in the area with water depth more than 400 mm, which has important guiding significance to the site selection of oasis development.

#### Temperature constraints of oasis distribution

**Figure 5:**
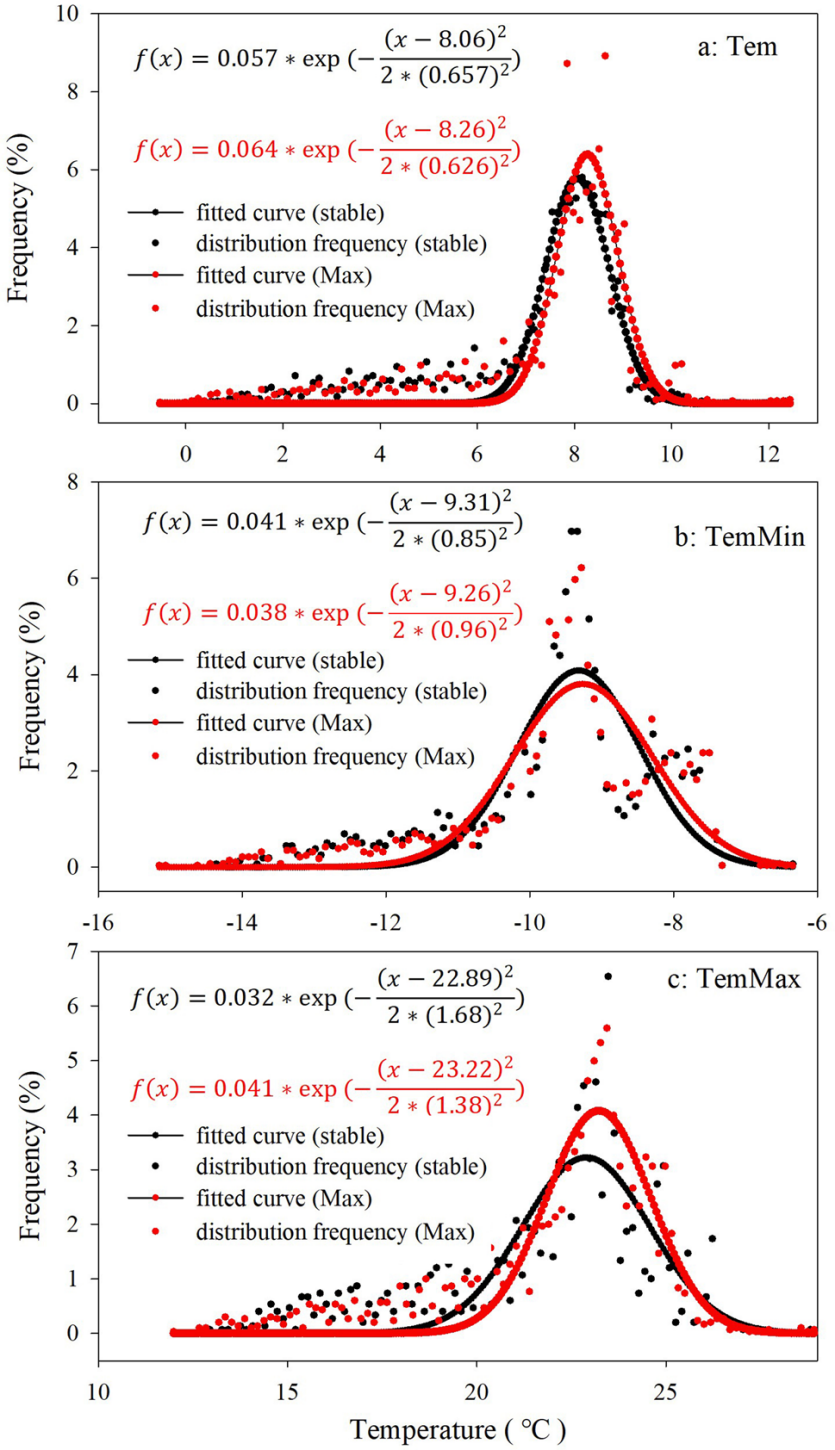
The distribution frequency of (**a**) mean temperature, (**b**) minimum temperature, and (**c**) maximum temperature in stable and Maximum oases (the legend is the same as Fig. 4).

Precipitation and air temperature are two primary climate parameters, which affect vegetation growth by changing soil moisture and heat energy, especially in arid-cold regions^28^. Using the mean, maximum, and minimum air temperature data, we examined how they are related to the distribution of oases. The results show that the oases distribution relate to mean air temperature ranges between 6 and 10 °C, with a maximum of around 7–9 °C (Fig. 5a), to minimum air temperature ranges between − 14 and − 7 °C, with a maximum of around − 10 to − 8 °C (Fig. 5b), to maximum air temperature ranges between 18–26, with a maximum of around 22–24 °C (Fig. 5c). Figure 5 also shows that oasis expansion occurs where the mean temperature, lowest temperature, and highest temperature are high. When the mean temperature is lower than 6 °C or higher than 10 °C, vegetation growth becomes restricted. The fitted normal distribution formulas of mean, maximum, and minimum temperature for stable and maximum oases were shown in Fig. 5, all the fits passed the test (*p* = 0.001).

### Driving force of oasis variation

The driving forces of oases variation were analyzed quantificationally based on the grey incidence model. The grey relative degrees (GRD) between oasis expansion and population, AWD, and GDP are pervasively high, and the general order is: rural laborer > total population > AWD > Primary industry > GDP > tertiary industry > secondary industry (Table 1).

**Table 1:** The relative degree of incidence between urban expansion and driving factors based on panel data in the Hexi Corridor (1986–2015).

Basins	Administration regions	GDP	Primary industry	Secondary industry	Tertiary industry	Per capita GDP	Total population	Rural laborers	Precipitation	AWD
SYRB	Liangzhou	0.6	0.64	0.59	0.63	0.62	0.98	0.99	0.91	0.9
	Jinchuan	0.58	0.71	0.56	0.61	0.61	0.94	0.92	0.82	0.77
	Yongchang	0.68	0.73	0.7	0.66	0.68	0.98	0.98	0.95	0.94
	Gulang	0.66	0.69	0.62	0.65	0.66	0.92	0.93	0.86	0.83
	Minqin	0.69	0.73	0.64	0.69	0.68	0.92	0.92	0.89	0.87
HHRB	Ganzhou	0.65	0.69	0.63	0.63	0.66	0.98	0.97	0.91	0.92
	Sunan	0.66	0.82	0.6	0.71	0.66	0.7	0.74	0.73	0.7
	Minle	0.66	0.72	0.61	0.65	0.66	0.96	0.97	0.95	0.96
	Linze	0.68	0.72	0.67	0.65	0.67	0.93	0.97	0.9	0.9
	Shandan	0.67	0.71	0.66	0.64	0.65	0.93	0.93	0.91	0.94
	Gaotai	0.69	0.75	0.65	0.67	0.69	0.95	0.98	0.91	0.93
	Jiayuguan	0.68	0.72	0.67	0.65	0.67	0.93	0.97	0.9	0.9
	Suzhou	0.67	0.71	0.66	0.64	0.65	0.93	0.93	0.91	0.94
	Jinta	0.69	0.75	0.65	0.67	0.69	0.95	0.98	0.91	0.93
SLRB	Guazhou	0.62	0.72	0.58	0.64	0.67	0.92	0.84	0.82	0.87
	Yumen	0.62	0.63	0.62	0.58	0.61	0.86	0.93	0.87	0.89
	Dunhuang	0.64	0.7	0.62	0.62	0.64	0.92	0.88	0.88	0.91

The GRD of Population, especially for the rural laborer, is the highest. The increase of the nonagricultural population directly stimulated urban residential, commercial, industrial, transportation, and other related industry development. Consequently, urban land expanded in this area. The population growth was a major factor in oasis variation^29^. During the past 30 years, the population increased from 1.06 to 5.07 million (378% increase), while the oasis area increased from 10,707 to 14,950 km^2^ (39.6% increase) in the Hexi Corridor. The rise in population will unavoidably lead to an increase in arable land for survival.

Secondly, the GRD between oasis expansion and AWD is pervasively high with the value around 0.9. The water resource including the precipitation and runoff play an important role in the spatial expansion of oasis. The Hexi Corridor located in a typical arid region, where the most vital limiting factor for both vegetation growth and economic development is the limited water resource. Concerning the shortage of water resources, it is difficult to irrigate many newly reclaimed agricultural oases in the Hexi Corridor. That is to say, water resources cannot afford continuous growth. Thus, AWD of oases is significantly positively correlated with the area of oases.

Thirdly, the GRD between economic factors, including GDP, Primary industry, Secondary industry and Tertiary industry, is about 0.6, which is relatively low comparing to that of population, water resource. The GRD of primary industry is highest with a value of 0.7 among the economic factors. Separately, for the type of agriculture oases contains most of the administration regions in the study area, the GRD of the primary industry was considerably higher than that of secondary and tertiary industry, the agriculture was their first driving force. For the resource-based cities and towns, like Jinchuan District, Jiayuguan City, the GRD of secondary and tertiary industry is essentially equal to that of primary industry, the secondary industry and tertiary industry played a vital role in oasis development.

## Discussion and conclusion

During the past decades, the oases in our study have gone through rapid changes, which is dominated by expansion^17,30^. The constraints of oasis distribution and the driving factors of oasis fluctuation are very complicated. Due, in part, to the difficulty of obtaining the fundamental data, quantitative description of the topography, hydrology, thermal constraints of the spatial distribution, as well as quantitative driving factors have been paid little attention^31,32^. Several attempts have been performed to analyze the oases expansions and their driving force in an arid region in northwest China^7,33,34^, but the constraints of oasis spatial distribution have been ignored. Thus, the present work fills this gap.

Many studies have qualitatively analyzed the driving forces of oasis expansion. Xie, et al.^32^ found that the dominant factors affected Jinta oases, which is a part of our study area, were the population growth and policy before 1980a, whereas the dominant driving forces were the changes in agricultural production ways and economic benefit and water utilization after 1980a. Zhou, et al.^19^ believed that the key driving force behind the oasis expansion in the Heihe river basin, the middle part of Hexi Corridor, was closely related to sufficient irrigation water guaranteed by excessive consumption of groundwater and surface water and water conservation efforts. Song and Zhang^31^ analyzed the driving forces in the Heihe River basin based on the random effects model and found that rural labor forces, annual temperature, and precipitation accelerated agricultural oasis variation.

According to the empirical analysis, most of the researches supposed that continuously enhanced human activities mostly driven the agricultural oasis expansion in the arid region of Northwest China. However, using a GRD model we found that the total population and rural laborer significantly accounting for the expansion of agricultural oasis in the Hexi Corridor. In addition, we also quantitatively examined the influence of driving forces on oasis expansion and the order of these factors is as follows: rural laborer > total population > AWD > Primary industry > GDP > tertiary industry > secondary industry. In addition, numerous policies including promoting economic development and restoring the ecological environment, conducted in this area from the 1980s contributed to the development of oasis.

The Household Production Responsibility System (HPRS) was implemented circa the 1980s in Hexi Corridor, which promoted the expansion of oasis in two ways: (1) greatly inspiring farmer’s production initiative which has resulted in an expansion of agricultural land, (2) releasing rural labor force from tilling the land to take up jobs in village-run factories and township enterprises, which have contributed to an increase in the use of human activities. The HPRS greatly emancipated and developed rural productive forces and promoted the growth of agriculture, which is a major driving factor for the expansion of oasis. At about the same period, the Construction of Commodity Grain Base (CCGB) was developed to improve crop production^35^. With a lot of steady and sustained investment in CCGB, the agricultural irrigation infrastructure, such as reservoirs, irrigation canals, water supply wells, and dams, was repaired or newly constructed. Since the 2000s, China’s ecological restoration and conservation policies, such as the ‘Three North Shelterbelt’, the ‘Grain for Green’, the ‘Natural forest protection’, and the ‘Comprehensive management plan for the ecological protection and construction’ of the Qianlian mountains’, have been proposed to protect the ecological balance and realize sustainable development. These projects, which are considered as ‘mega-engineering’ activities and the most ambitious afforestation and conservation projects in human history^36^, compensated participating farmers for converting their cropland back to forest or grassland with a cash or grain subsidy and prohibited the disorderly development of ecological land. In summary, Numerous policy factors conducted in this area also contributed to the development of oasis.

Generally, the oases location and layout in arid land were influenced by the spatial distribution of water resources, the main driving forces of oasis spatial expansion in the arid region were population, water resource, economy, national policies, and other factors.

Although the current study represents constraints and driving forces of oasis development in Hexi Corridor, it is important to keep in mind that our work necessitated some improvement. The AWD has been used to depict the hydrology restriction of oasis distribution and the driving force of oasis expansion, the exact amount of water resources including surface water, underground water, and rainfall were not sufficient to analyze the water resource in Hexi Corridor. The driving forces of oasis develop was rather complex, including the effects of human activity and climate change, and their overlapping effects^37^. However, the GRD model is powerful in estimating the influences of factors, but the interactions between the influencing factors remain indistinguishable. Geomorphological attributes have effects on the distribution of oases especially in areas of rough topography^26^. The slope aspect is a landscape feature identified as a key factor driving spatial differences in the ecological and structural characteristics of vegetation. Other elements of topography, such as slope gradient, elevation, influence the oases distribution at least through surface and subsurface redistribution of water potentially available to plants. Microtopography plays an important role in analyzing the constraints of oasis distribution. DEM data adopted in present work, 90 m spatial resolution, may erase part of the microtopography information. Despite these limitations and uncertainties, the present approach is adequate for the first time to better understand the constraints of oasis development and driving forces of the oasis expansion in the arid land.

In this study, the oases changing data over a long period from 1985–2015 in the Hexi Corridor were used to analyze the constraints and driving forces. The topography, hydrology, and heat restrictions were analyzed, and the driving forces of oasis expansion were assessed quantitatively through the GRD model. The outcomes from this study may have significant implications for contributing to better management and planning of oasis development in the arid land.

In conclusion, a clear understanding of the relationship between oasis change and their constraints and driving forces is of great significance to sustainable management and development of the oasis landscape in arid areas. The oasis area in the Hexi Corridor expanded 40% from 1986 to 2015, the stable oasis area was 9062 km^2^, while the maximum area reached 16,374 km^2^. The constraints for oases of topography, hydrology and heat condition play an important in oasis development. The main driving forces of oasis spatial expansion in the arid region were population, water resource, economy, policies, and other factors. Moreover, the proposed results are expected to contribute to optimizing oasis development and minimizing environmental impact.

## Study area and methods

### Study area

Hexi Corridor is a long and narrow passage, with a width of 40–100 km (north–south) and a length of 1120 km (east–west), and with an area of 27.6 × 104 km^2^. This region is characterized by a typical temperature desert climate, i.e., dry with very litter annual precipitation no more than 200 mm that mainly occurs in summer, plenty of annual potential evaporation between 1500 to 3200 mm.

**Figure 6:**
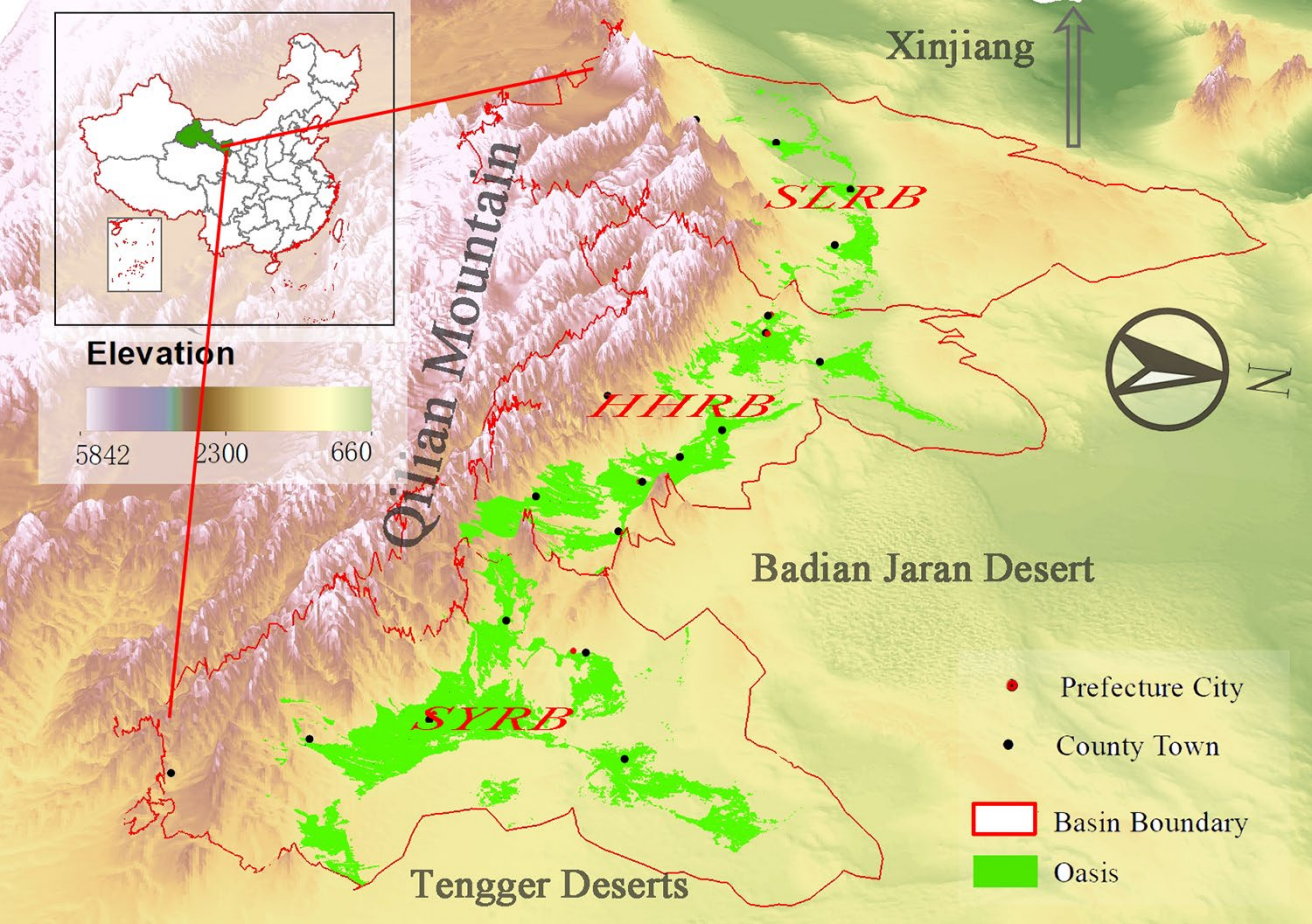
The oasis distribution in Hexi Corridor in 2015. The map was generated using ArcGIS 10.3, www.esri.com.

There are three independent landlocked river basins from east to west, i.e., SYRB, HHRB, and SLRB, as illustrated in Fig. 6. All the upstream of the three river basins are the Qilian mountain; this study focused the oases lied in middle and lower reaches. There are 17 administrative regions in middle and lower reaches, respectively. The administration regions in the middle and lower reaches and the rivers in each river basin are shown in Table 2. The runoff from these rivers is measured by hydrologic stations (marked in brackets), as illustrated in Table 2.

**Table 2:** The administrative regions in the middle and lower reaches of three river basins in the Hexi Corridor.

River basins	Parts of the basin	The administration regions	Main rivers flow into this area (hydrographic station for this river)
SYRB	Middle reaches	Liangzhou District, Jingchuan District, Yongchang County, Gulang County	Dajing River (Dajin reservoir), Gulang River (Gulang), Huangyang River (Huangyang reservoir), Zamu River (Zamusi), Jinta River (Nanying reservoir), Xiying River (Jiaotiaoling), Dongda River (Shagousi), Xida River (Xida reservoir)
	Lower reaches	Minqin County	
HHRB	Middle reaches	Ganzhou District, Linze county, Shandan County, Minle County, Sunan County, Suzhou District, Gaotai County, Jiayuguan City,	Beida River (Binggou) and Hehei River (Yingluoxia);
	Lower reaches	Jinta County	
SLRB	Middle reaches	Yumen City, Dunhuang City,	Shule river (Changmapu), and Dang river (Dangchengwan)
	Lower reaches	Guazhou County	

Administrative regions are county-level administrative regions by default.

### Data source

The LULC datasets of the study area, including oases and deserts in 1986, 1990, 1995, 2000, 2005, 2010, and 2015, were derived from Landsat TM\ETM\OLI imagery. Oases include the LULC types regarding vegetation, water areas, residential areas, and industrial and mining land, while deserts constitute the remaining LULC types^30^. The overall accuracy values (kappa coefficient) of the seven periods are above 0.79; the classification results are therefore reliable and suitable for oasis change analysis. More details about oasis extraction and its accuracy can be found in this literature^30^.

The elevation and slope were calculated from SRTM DEM with a spatial resolution of 90 m downloaded from the USGS website. The raster-based precipitation and temperature data from 1986 to 2015 were collected from the 1 km monthly temperature and precipitation dataset^38^ (available at https://doi.org/10.5281/zenodo.3114194 for precipitation and https://doi.org/10.5281/zenodo.3185722 for air temperatures), which were created by spatially downscaling with the resolution of 0.5 arcminutes (~ 1 km). The socio-economic data including population, income, grain yield, and labor for each administration region were obtained from local statistic yearbook 1985–2015 (each yearbook every five years) (Gansu bureau of statistics). Water resource data (the runoff volumes of the headstreams) from 1985–2015 were acquired from the Annual Report of Water Resources in Gansu Province (https://slt.gansu.gov.cn).

### Constraints for oasis development and driving forces of oasis variation

Oasis distribution in the arid region results from the natural adaptability factors and is affected by environmental conditions^26^. Quantifying evaluation of the relationship between the distribution of oases in arid land and its constraints is significant for managing the oases development. The topography and landforms^39,40^, available water resource^41–43^, and temperature^44^ (Table 3), which were critical for the formation and evolution of oasis, were used to depict the range of oasis distribution by the spatial analysis of geographic information system.

**Table 3:** Indicator system on constraints and driving factors of oasis variation. Statistics and analysis of constraints are carried out on the scale of the grid with 90 × 90 m, and analysis of driving factors are carried on the scale of county-level administrative units.

Constraints		Symbol	Driving factor		Symbol
Topography and landforms	Elevation	DEM	Population	Total population	P_t_
	Slope	Slope		Agricultural population	P_a_
Water resource	Precipitation	P	Natural factor	Accessibility water depth	AWD
	Accessibility water depth	AWD	Economic factor	GDP	GDP
Temperature	Average air temperature	T_avg_		Added-value of agriculture	GDP_1_
	Maximax temperature	T_max_		Added-value of industry and construction industry	GDP_2_
	Minimax temperature	T_min_		Added-value of commercial and service industry	GDP_3_

We posit that the oasis stable area where the oasis remains stable, never changed in a long time, is an ideal place for oasis development and could provide good conditions including topography, climate, and water resources. Furthermore, the maximum oasis area where the oasis occupied at least once, is a suitable place for oasis development. It is noteworthy that the stable oasis area has very favorable location conditions and can resist the interference of nature and human activities. The maximum oasis area also has good hydrology, climate, and geomorphology conditions, which is the place where oasis was developed. Base on the oasis stable area and maximum area extracted from oasis distributions in seven periods by superposing together, superposition analysis of oasis area and limiting factors was conducted to obtain the constraints of the oasis in the arid land.

However, the spatial expansion or shrinkage resulted from external driving factors including population increase, economic development, industrial adjustment, and water resource variation. To quantificationally represent the driving force of oasis area variation, we chose the oasis area as an independent variable and chose the natural elements, social and economic factors as the dependent variable. An indicator system on driving factors was built up (Table 3). The grey incidence method was used to analyze the driving forces of oasis variation, and the calculation steps was in the literature^45^.

Accessibility water depth (AWD) was built based on the available runoff (R) and precipitation (P). The available runoff on grid-scale was the average amount of the upstream runoff on its irrigated oases, which is measured at the mountain pass hydrologic station. The gridded precipitation data (P) were derived from gridded precipitation data mentioned above. The AWD based on the grid was the sum of R and P, representing the water resources that can be used in the oasis grid.
